# A Methodological Approach to Measuring the Impact of TAK-003 for the Prevention of Dengue in Dourados, Brazil: Optimizing Strategies for Public Health

**DOI:** 10.3390/vaccines13020121

**Published:** 2025-01-25

**Authors:** Benedetta Ghezzi, Cristina Valencia, Roberto Dias de Oliveira, Daniel Tsuha, Waldno Lucena Júnior, Alberta Di Pasquale, Morgan Mc Namara, Juliana Senra, Denise Abud, Julio Croda

**Affiliations:** 1 Takeda Pharmaceuticals International AG, 8152 Zürich, Switzerland; 2 Oswaldo Cruz Foundation, Federal University of Mato Grosso do Sul, Campo Grande 79070-900, MS, Brazil; 3 Nursing Course, State University of Mato Grosso do Sul, Dourados 79804-970, MS, Brazil; 4 Municipal Health Department of Dourados, Municipal Prefecture of Dourados, Dourados 76051-053, MS, Brazil; 5 Takeda Pharmaceuticals International AG, Singapore 018981, Singapore; 6 Takeda Pharmaceuticals International AG, São Paulo 04794-000, SP, Brazil

**Keywords:** dengue, vaccination, public health, methodology, TAK-003

## Abstract

**Background/Objectives**: Takeda’s tetravalent dengue vaccine TAK-003 has been approved by the Brazilian regulatory agency ANVISA for dengue disease prevention in individuals aged 4 to 60 years. Dourados, in the state of Mato Grosso do Sul, became the world’s first city to implement a mass vaccination campaign targeting approximately 120,000 individuals. An ongoing collaborative, observational, population-based study using national surveillance and vaccination data was planned to measure the impact of the vaccine on the reduction in dengue incidence. **Methods**: In this manuscript, the study’s methodology, including its programmatic steps and public health relevance, is described. A collaborative assessment with multidisciplinary researchers in Brazil was conducted to identify key programmatic areas for the successful implementation of the study. These areas included feasibility and site selection assessment, methodology selection, vaccination program implementation, and public health importance. **Results/Conclusions**: Identification of the public health problem and understanding the disease burden, local healthcare infrastructure, and strategic partnerships were critical for a robust feasibility assessment. One of the feasibility criteria identified was the ability of the Dourados Municipal Health Secretary and the principal investigator to conduct an active vaccination campaign, utilizing extramural activities and diverse communication channels to increase vaccine acceptance and coverage. The selection of analytical methods, such as time series analysis, was dependent on the national and local structures of the databases and data availability.

## 1. Introduction

Dengue virus (DENV) is a species within the genus Flavivirus and family Flaviviridae of enveloped viruses with non-segmented positive-sense RNA genomes; there are four recognized DENV serotypes, DENV-1, DENV-2, DENV-3, and DENV-4 [1]. The transmission of the virus into and through the human population primarily occurs through the bite of an infected mosquito from the Aedes genus, typically *Aedes aegypti* [2]. Dengue infection can result in a wide range of symptoms from mild/moderate dengue fever to life-threatening dengue hemorrhagic fever/dengue shock syndrome [3,4].

In the Americas, dengue has an endemic–epidemic pattern, experiencing outbreaks every 3 to 5 years. In the past decade, the burden of dengue in Brazil has increased rapidly to a hyperendemic scenario, with all four DENV serotypes circulating since 2010 [5,6]. In 2023, an incidence of 773.1 cases per 100,000 people was reported in Brazil [7], of which 24,429 were dengue cases with warning signs [8,9], with most cases occurring in the midwest and south regions. The municipalities with the highest reported incidence rates were Goiânia (3632.1 cases per 100,000 people) [8,9], followed by Brasília (2283.9 cases per 100,000 people). In 2024, there were substantial increases in dengue incidence across Brazil, with an overall incidence rate of 3112.8 cases per 100,000 people and a total number of 84,540 cases of severe dengue with warning signs. The municipalities with the highest dengue incidence rates in 2024 were Brasília (9657 cases per 100,000 people) and Minas Gerais (8166.9 cases per 100,000 people) [10]. Historically, Brazil has grappled with dengue epidemics caused primarily by the DENV-1 and DENV-2 serotypes. However, in 2023, a significant shift in this pattern was observed, as DENV-3 had a notable resurgence [11].

Dourados, a municipality in the state of Mato Grosso do Sul, Brazil, is the second-most populous in the state and has experienced epidemic cycles of great impact over the years. The environmental conditions (temperature between 20 °C and 40 °C, moderate or high relative humidity, and high average rainfall) between November and April are critical for the maintenance of dengue epidemics. Preventative measures, such as widespread vaccination and vector control, are essential to reduce infection, hospitalization, and death.

Takeda’s tetravalent dengue vaccine TAK-003 (Qdenga^®^) is a live-attenuated tetravalent dengue vaccine based on a DENV-2 backbone, with three additional recombinant viruses containing the DENV-1, -3, and -4 components. TAK-003 was evaluated in a long-term efficacy trial in children and adolescents living in dengue-endemic areas of Asia and Latin America. After 4.5 years, the cumulative vaccine efficacy against symptomatic dengue was 61.2%, and efficacy against dengue leading to hospitalization was 84.1% [12]. Further exploratory subgroup analyses demonstrated long-term efficacy and safety against all four DENV serotypes in previously exposed individuals and against DENV-1 and DENV-2 in dengue-naïve individuals [12]. The vaccine regimen consists of two subcutaneous injections of 0.5 mL TAK-003, administered 3 months apart, without pre-vaccination screening. On 2 March 2023, TAK-003 was approved for the prevention of dengue disease in individuals aged 4 to 60 years by the Brazil’s National Health Surveillance Agency (ANVISA).

On 3 January 2024, approximately 10 months after TAK-003 approval by ANVISA, Dourados in the state of Mato Grosso do Sul became Brazil’s first municipality to launch a mass vaccination campaign targeting approximately 120,000 individuals between the ages of 4 and 60 years [13]. To measure the impact of the vaccine in the reduction in DENV incidence, an ongoing collaborative, observational, population-based study using national surveillance and vaccination data is being funded by Takeda. Data from this study are currently being collected, and the results of the analysis will be reported upon completion of the post-vaccination period (2025–2027). In this manuscript, we describe the study methodology, including the programmatic steps and public health relevance. Although this study does not aim to evaluate the ongoing vaccination program, the experience is described.

## 2. Materials and Methods

We consulted dengue research experts in the municipalities of Dourados and Ponta Porã (given the similar profiles of DENV risks and transmission patterns) on the study’s feasibility, selection of methodology, and public health relevance. Dengue research experts were also consulted on the communication strategy and overall vaccination program established by the Dourados Municipal Health Secretary.

### 2.1. Assessment of Feasibility

In 2023, a feasibility assessment was performed to determine the quality, reliability, and accessibility of data source(s) available to measure the impact of TAK-003 on dengue incidence in Brazil following the vaccination program.

**Figure 1:**
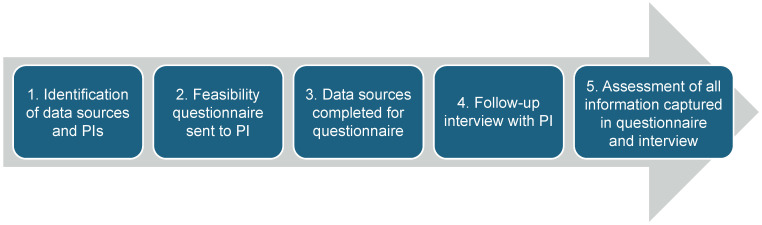
Feasibility assessment process steps.

This feasibility assessment (Figure 1) included a literature review, dengue research expert questionnaire development and implementation, and interviews. The literature review consisted of a report search from 2019 to 2023 on dengue epidemiological measures in Brazil, including but not limited to incidence rates, mortality, and transmission dynamics. Eleven reports were identified in total, which supported the identification of potential study settings, data sources, and principal investigators (PIs). Criteria for the feasibility assessment questionnaire were defined with input from the Takeda Brazilian local team, and questions addressed the following domains: data relevance, representativeness, data quality, accessibility, and general database characteristics. The questionnaire was sent to potential PIs (self-administered) around the country; three identified PIs that returned the questionnaire then took part in interviews (which took the form of meetings and conversations) to answer additional questions. The unstructured interviews assessed the PIs’ knowledge of the field, capacity, and professional network, in addition to the infrastructure, epidemiology, and public health context for vaccine implementation in their geographic working areas. A final assessment of all of the information captured in the questionnaire and interviews was conducted to select the PI and setting to be used for the study.

As part of this assessment, we describe the ongoing vaccination program in the Dourados municipality to provide a comprehensive understanding of the context in which the study is conducted and its public health relevance.

### 2.2. Definitions and Classification of Dengue Cases

Since 2014, Brazil has used the following classifications for cases of the disease: dengue, dengue with warning signs, and severe dengue. The classifications are made using the patient’s clinical, laboratory, and epidemiological information [14].

A laboratory-confirmed case of dengue is defined as any suspected case that has been confirmed by one or more of the following laboratory tests and their respective results: non-structural protein 1-antigen enzyme-linked immunosorbent assay (ELISA), positive viral isolation, detectable reverse transcription polymerase chain reaction (up to the 5th day after the onset of symptoms), ELISA immunoglobulin M antibody detection (from the 6th day of the onset of symptoms), a 4-fold increase in antibody titers in the plaque reduction neutralization test or hemagglutination inhibition test using paired samples (acute and convalescent phases). Serological diagnosis should include an evaluation of cross-reactivity with other flaviviruses, and confirmation should include a test for differential diagnosis for other diseases. After laboratory verification of the viral circulation in the area, confirmation is carried out based on clinical–epidemiological criteria.

Suspected cases of dengue fever can correspond to an individual who lives in an area where dengue cases are recorded or an individual who has traveled, in the past 14 days, to an area with dengue transmission (or presence of *Aedes aegypti*). The individual must have a fever (usually lasting between 2 and 7 days) and present with two or more of the following alarming signs: myalgia, arthralgia, headache, retro-orbital pain, nausea, vomiting, rash, petechiae, positive loop test, and leukopenia. For children, a suspected case corresponds to a child from (or resident in) an area with dengue transmission with an acute febrile condition, usually lasting between 2 and 7 days, and without signs and symptoms indicative of another disease.

A suspected case of dengue with warning signs is a case of dengue that, during the period of defervescence of the fever, presents with one or more of the following alarming signs: intense and continuous abdominal pain or pain on palpation of the abdomen, persistent vomiting, accumulation of fluids (ascites, pleural effusion, or pericardial effusion), mucosal bleeding, lethargy or irritability, postural hypotension and/or lipothymia, hepatomegaly > 2 cm, and progressive increase in hematocrit.

A suspected case of severe dengue is a case that presents with one or more of the following alarming signs: shock due to severe plasma extravasation evidenced by tachycardia, cold extremities and a capillary refill time ≥3 s, a weak or undetectable pulse or convergent differential pressure ≤20 mmHg, late-stage arterial hypotension, fluid accumulation with respiratory failure, and severe bleeding according to physician assessment (hematemesis, melena, massive metrorrhagia, and central nervous system bleeding).

### 2.3. Selection of Analytical Methodology

**Figure 2:**
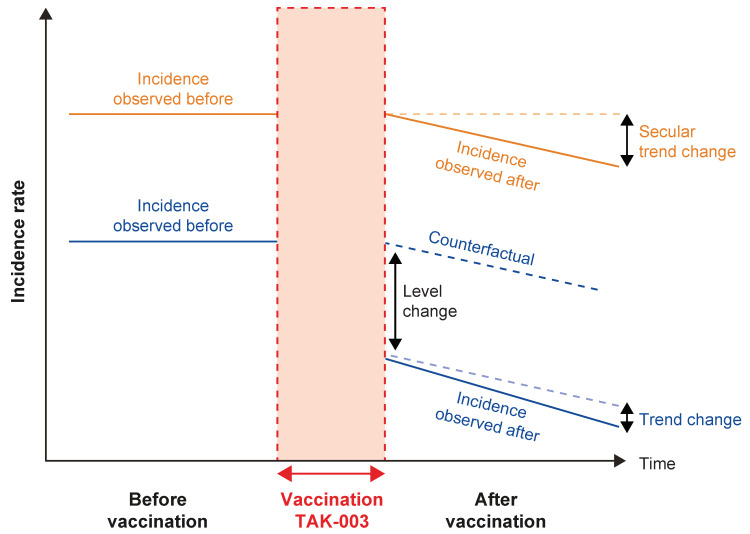
An overview of the controlled interrupted time series design. The target population is indicated in blue, and the control population is indicated in orange. Secular change refers to the long-term trend or pattern in disease incidence over an extended period, the counterfactual is the hypothetical scenario in which the target population did not receive the vaccine, level change refers to a sudden and significant shift in the measurable outcome, and trend change refers to a shift in the rate of change of a measurable outcome.

An interrupted time series (ITS) and a controlled ITS (CITS) design (Figure 2) were chosen to measure the overall effect of vaccination in the Dourados population receiving the TAK-003 vaccine [15]. The Ponta Porã municipality was used as a control population, where the vaccine was not available at the time of region selection. The CITS design is a suitable quasi-experimental design to evaluate the population-level impact of an intervention (such as vaccination) and carries several advantages: there is no need for individual-level data, the direct and indirect effects of vaccination can be estimated, and a control population analysis permits adjustment for unmeasured time-varying factors [15].

The use of a control is a requirement of the feasibility assessment, and Ponta Porã was used as a comparator (control) group to strengthen the study design. This population did not receive the vaccine but has similar dengue risk and transmission patterns. In 2023, there were 3378 cases of dengue reported in Dourados, and there were 3653 [10] cases reported in Ponta Porã. Over the years, both Ponta Porã and Dourados have experienced recurring dengue epidemics with comparable cycles. This shared epidemic pattern strongly suggests that environmental factors, particularly the climate, play a dominant role in influencing the onset and intensity of dengue outbreaks in these regions, and they are similar between the two municipalities [16,17]. The use of a control provides a greater ability to infer causality by isolating the effect of vaccination. The analyses also considered independent factors that may affect dengue incidence in both Ponta Porã and Dourados, including political, climatic, and social factors, or the overall transmission pattern.

Data were extracted and aggregated from the Brazilian public health surveillance databases to look at dengue incidence over time, before (2013–2023) and after (2025–2027) the vaccination program. Disease counts and vaccine effect are assessed at the population level in real-world conditions. Population refers to the age group, and outcome measures are the cases of dengue reported in the population before and after the vaccination program. Average incidence and trends over time (increasing and decreasing) and counterfactual comparisons (i.e., expected in the absence of intervention) were considered.

During the TAK-003 vaccination program, assumptions about dengue incidence rates include a gradual decline in incidence as vaccine coverage builds, potentially uniform effects for modeling simplicity, and the possibility of delayed effects, requiring careful consideration of lagged impacts.

## 3. Results

### 3.1. Assessment of Feasibility

#### 3.1.1. Country and Setting Selection

Two study settings were initially considered: Dourados in Mato Grosso do Sul and Maré in Rio de Janeiro. Maré is a favela settlement that presented multiple logistical challenges that could negatively impact data quality; the key issues included a low number of cases, a weak surveillance structure, and high underreporting. Dourados is the second-most populous municipality (243,367 inhabitants) in the state of Mato Grosso do Sul, and it has a vulnerable population, with one of the largest indigenous reserves in Brazil. The municipality has robust epidemiological surveillance, with high rates of laboratory confirmation of suspected cases (virologically confirmed) and historical data from 10 years of registration of dengue cases. The Dourados Municipal Health Secretary uses the electronic system e-SUS PEC to record data on doses of TAK-003 administered and the vaccinated population.

Ponta Porã, also in the state of Mato Grosso do Sul, is a municipality located approximately 100 km from Dourados, with a smaller population of 92,017 individuals. Ponta Porã was selected as a control population due to having a similar profile regarding dengue risks and transmission patterns, age distribution, and vector control measures.

**Figure 3:**
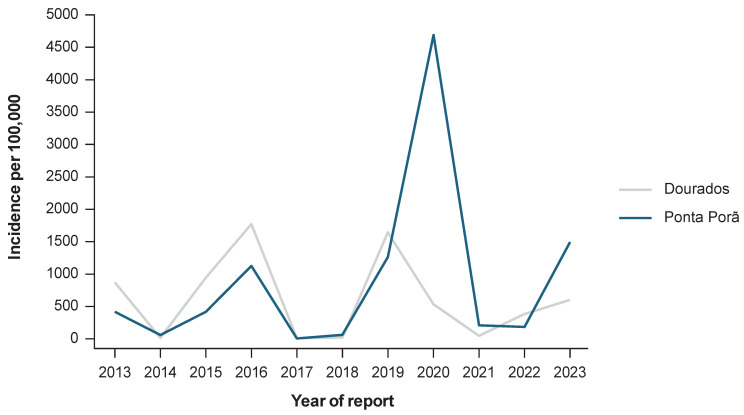
The incidence of confirmed dengue cases in the Dourados and Ponta Porã municipalities for the entire population between 2013 and 2023 [18].

Epidemiological data from 2023 (extracted from the Brazil Notifiable Diseases Information System [SINAN]) reported 3378 confirmed cases of dengue in Dourados, while there were 3653 cases reported in Ponta Porã (Figure 3) [18]. In 2023, Dourados had a dengue incidence rate of 1388 per 100,000 people, while Ponta Porã had an incidence rate of 3970 per 100,000 [19,20]. Over the years, both Ponta Porã and Dourados have experienced recurring dengue epidemics with comparable cycles. Both Ponta Porã and Dourados implement weekly larval inspections and monthly ultra-low-volume (ULV) insecticide spraying campaigns as part of the Mato Grosso do Sul state dengue control program. In addition, educational campaigns for reducing standing water are conducted during peak transmission months (November to March) in both locations. The similar age distribution indicates a comparable demographic structure relevant for assessing dengue susceptibility.

In summary, the selection of the municipalities of Ponta Porã and Dourados as study settings was based on their shared similarities regarding dengue risk, transmission patterns, surveillance system, vector control measures, and age distribution.

#### 3.1.2. The Selection of the Principal Investigator

PIs from two different Brazilian states, Rio de Janeiro and Mato Grosso do Sul, were identified as potential collaborators. The PI from Mato Grosso do Sul was selected due to his experience with previous impact studies and vaccination programs, access to different national and local databases needed to perform the study, and his ongoing collaboration with the Dourados Municipal Health Secretary.

#### 3.1.3. Burden of Dengue Disease and Available Data Sources

When assessing the burden of dengue disease and availability of data sources, the municipalities of Dourados and Ponta Porã both reported high dengue incidence: Dourados reported 6830 confirmed cases, and Ponta Porã reported 9922 between 2013 and 2023 for the entire population (Figure 3). There was a high rate of data completion and accessibility to data sources, with the data field “onset of symptoms” having 100% completeness. Furthermore, there were only 11 (Dourados) and 45 (Ponta Porã) records with missing information, but all age-related information was complete. The rates of completeness for confirmation criteria for both municipalities were as follows: Dourados had 15,714 of 22,650 (69%) cases confirmed by a laboratory and Ponta Porã had 6935 of 17,214 (40%) cases confirmed.

#### 3.1.4. The Vaccination Program

The Dourados vaccination program, led by the municipal health department, began on 3 January 2024, with an aim to vaccinate up to 120,000 residents aged 4 to 60 years before the next dengue-endemic season (March–May) [21]. It is important to note that the start of the National Immunization Program (NIP) for adolescents (aged 10–14 years) occurred 1 month after the Dourados program had started. The NIP’s implementation was unplanned at the time of our study’s inception. Between 3 January and 31 July 2024, 88,000 people were vaccinated with at least one dose in Dourados, and more are expected to be vaccinated in subsequent months as the program continues. The Dourados Municipal Health Secretary had 33 equipped health units carrying out the vaccination program. These units were solely and fully responsible for the program in accordance with standard procedures for communicating to and recruiting the eligible population, as well as for the storage and application of vaccines. The program required a multifaceted approach to maximize vaccine uptake, and its success depended on the level of acceptance of the vaccine to achieve high coverage rates.

Having an established and defined communication strategy, which included the use of media outlets for information dissemination, was essential to generate awareness and educate the population. The importance of targeted communication strategies for specific groups, such as children and the elderly, and the cost-effective use of local infrastructure to support program implementation became evident and were pivotal in the rollout of the vaccination campaign. A key finding was that extramural activities and public awareness campaigns can be used, alongside school education programs, to boost target vaccination coverage; this can include partnerships with universities, supermarkets, gym centers, shopping malls, and charities. Establishing setting-specific measurable goals for vaccine coverage and disease reduction was also identified as a key objective of the vaccination campaign, in parallel with the establishment of a strong and reliable supply chain for vaccine procurement, storage, and distribution.

The vaccination program provided easy accessibility to vaccines in both urban and rural areas, in addition to the training of healthcare providers to administer vaccines according to the approved label. It also supported the establishment of accessible vaccination centers, including hospitals, clinics, schools, and mobile units. Community leaders, healthcare workers, and organizations were also involved in promoting vaccination and establishing trust within the community through transparency and addressing concerns respectfully during the progression of the campaign. This was complemented with the robust implementation of systems to monitor vaccine coverage.

Lastly, ensuring strong political will and commitment to support vaccination efforts, and the establishment of strong leadership and management teams to provide oversight, were critical to the implementation of the program.

### 3.2. Selection of Methodology

The ITS and CITS study designs were used to measure the impact of the vaccination program [15]. The ITS study design was considered because it is one of the most rigorous quasi-experimental designs to assess the longitudinal effects of interventions. The ITS design uses a negative binomial regression model with the monthly incidence of cases as an outcome; this model estimates impact and is adjusted for seasonality and autocorrelation. The CITS is a version of the ITS that uses both absolute incidence and trends in the control population to estimate the impact of the vaccination program on the population. In this case, the CITS allows for an estimate of the impact of the vaccination program in Dourados by using the absolute incidence and trend observed in Ponta Porã (control population). Descriptive outputs from the CITS analysis include dengue frequency and incidence tables. The incidence rates will be expressed as cases per 100,000 person-years. Coefficients, with their 95% confidence intervals, from the fitted regression model will permit an inference to be made regarding the impact of the vaccination program in Dourados on dengue incidence. Incidence rate ratios (IRRs) will also be calculated by dividing the incidence rate in the post-vaccination period by that in the pre-vaccination period. Vaccine impact will be calculated as (1 − IRR) × 100 and will be expressed as a percentage.

## 4. Discussion

This manuscript describes the programmatic steps and public health relevance of conducting a collaborative, observational, population-based study using national surveillance and vaccination data to measure the impact of vaccination on the reduction in dengue incidence in Brazil following a vaccination program. A number of lessons were identified: the identification of Dourados and Ponta Porã as the study and control populations, respectively, was a critical first step. The key criteria considered during the step included the geographic distance between both populations, age distribution, dengue risk profile, and transmission patterns, as well as vector control measures and surveillance systems. The similarities between Dourados and Ponta Porã in these key areas supported their inclusion in this study. Following site selection, the selection of a PI with relevant experience in the implementation of impact studies and vaccination programs was essential. In addition to experience, it was crucial that the PI had access to the required national and local databases.

When assessing the burden of dengue disease and the availability of data sources, the municipalities of Dourados and Ponta Porã both reported a high dengue incidence between 2013 and 2023 for the entire population, which was sufficient to justify their inclusion [20]. However, the incidence of dengue during the 2020 peak was higher in Ponta Porã [18]. Data completion and the accessibility of data sources were important prerequisites that were satisfied by both municipalities; few records had missing information and rates of completion for virological confirmation criteria were acceptable.

The identification of the correct analytical methods given the available data was critical in the study design stages, with the CITS design ultimately being chosen due to the use of a control to strengthen the analysis. Furthermore, the CITS design has several key advantages: there is no need for individual-level data, the direct and indirect effects of vaccination can be estimated, and a control population analysis permits adjustment for unmeasured time-varying factors [15]. It is also important to note that the CITS method does not assume a parallel trend; differing trends between the two municipalities can be easily modeled, avoiding bias in the estimates. Therefore, the CITS approach will provide the best interpretation of the observed data in both the intervention and control populations.

In both the Dourados and Ponta Porã municipalities, there was a large proportion of the population with limited income and access to information on the dengue vaccination and where to go to receive it. Communication strategies would be needed to target this group, as dengue disease is not perceived as a risk; this could be addressed by raising awareness through the radio and social media, as television is not generally of interest [22].

Identifying an appropriate time to start the vaccination program to promote uptake is critical. The vaccination program reported here started in January 2024, with the objective being to vaccinate the target population before the next dengue-endemic season; however, the program also coincided with summer holidays and the Carnival of Brazil. This created a narrow period to complete the vaccination program before the holidays finished and children/students returned to school. Furthermore, the start of the National Immunization Program (NIP) for adolescents (aged 10–14 years) was 1 month after the Dourados program had started. This created confusion regarding vaccine eligibility, as the NIP only targeted adolescents and not the broader age group [23]. The NIP implementation was unplanned at the time of our study’s inception; however, it is acknowledged that such changes in the vaccination program are expected in a prospective study. Any changes in the vaccination program will be accounted for during the analysis of the study results.

Another consideration is the speed required to reach the ideal vaccination coverage of approximately 60% of the overall population aged 4 to 60 years. It is preferable to recruit the target population in a relatively short period of time and before the start of the next dengue-endemic season, as this helps to ensure a more homogeneous epidemiological baseline.

This methodological assessment highlights the importance of building effective partnerships with various organizations, including universities, gym centers, and charities, to help raise awareness of dengue and highlight the importance of vaccination, both inside and outside of the classroom.

Accessibility is a key factor to address through community outreach, as it was noted in the study that some individuals do not wish to (or cannot) go to health units to be vaccinated. Therefore, vaccination programs should try to reach these individuals at their location. It is usually easier to target students and workers for vaccination, as they are more mobile than home-based demographic groups. For those in employment, workplace vaccination drives can further boost vaccine uptake. Interestingly, vaccine skepticism for the dengue vaccine was not as pronounced as that noted for other vaccines; consequently, there is generally a willingness to be vaccinated if individuals receive assistance to access the vaccine and/or vaccination centers [24].

The selection of the ITS and CITS study design approaches poses several limitations, including the risk of infection and disease being the same for the population at both time periods (before and after intervention) [15]. This is not always possible because dengue epidemics can affect populations rapidly, and seasonal patterns will also vary the risk of infection for a given population over time; the design cannot control for these concurrent factors or events affecting trends. Furthermore, the study can be influenced by population behavior changes resulting from economic and political changes (international or national) and awareness campaigns.

The study design also limits the ability to test for statistical associations and make conclusions on causality. Such limitations include a reliance on existing surveillance systems (which limits the ability to control data quality and completeness) and the collection of aggregated data only, preventing individual-level inferences. This study’s exclusive use of aggregate-level data also means it cannot provide information about effectiveness at the individual level.

This assessment has demonstrated the need to have a thorough feasibility assessment, including an understanding of the correct analytical methodology needed to structure and implement a vaccination study that focuses on measuring the impact of the vaccination program on dengue burden in an endemic setting. In addition, it is important to consider that the ability to monitor the impact of such a vaccination program is dependent on various operational factors, such as the capacity of the surveillance system, the data reporting quality, the operational effectiveness, and the vaccine acceptance of the local population, which may be different in other municipalities. The methodology described in this publication is substantiated solely for the purpose of measuring the impact of the TAK-003 vaccination program on the reduction in dengue incidence as part of the ongoing observational, population-based study. Therefore, the integration of this approach into epidemiological monitoring and infectious disease control programs has not been considered.

## 5. Conclusions

The use of a robust feasibility assessment is crucial to identify public health problems, understand disease burden and local healthcare infrastructure, and build strategic partnerships. The tripartite collaboration between the PI, Takeda, and the Dourados Health Authorities/municipality was critical for successful study implementation. Furthermore, the selection of appropriate analytical methods is dependent on national and local database structures, data quality, and accessibility. In the case of Dourados, accessibility was improved through the efforts of the local health department to enhance communication campaigns. The aim of this targeted approach is to boost both vaccination coverage and population-level uptake.

## Data Availability

No new data were created or analyzed in this study. Data sharing is not applicable to this article.
